# Chemokine Receptors CCR1 and CCR2 on Peripheral Blood Mononuclear Cells of Newly Diagnosed Patients with the CD38-Positive Chronic Lymphocytic Leukemia

**DOI:** 10.3390/jcm9072312

**Published:** 2020-07-21

**Authors:** Irina Kholodnyuk, Alla Rivkina, Laura Hippe, Simons Svirskis, Svetlana Kozireva, Ildze Ventina, Irina Spaka, Marina Soloveichika, Jelena Pavlova, Modra Murovska, Sandra Lejniece

**Affiliations:** 1 Institute of Microbiology and Virology, Riga Stradins University, Riga LV-1067, Latvia; Laura.Hippe@rsu.lv (L.H.); Simons.Svirskis@rsu.lv (S.S.); Svetlana.Kozireva@rsu.lv (S.K.); ildze.ventina@gmail.com (I.V.); irina.spaka@inbox.lv (I.S.); Jelena.Pavlova@rsu.lv (J.P.); Modra.Murovska@rsu.lv (M.M.); 2 Department of Internal Diseases, Riga Stradins University, Riga LV-1038, Latvia; Alla.Rivkina@rsu.lv (A.R.); Sandra.Lejniece@rsu.lv (S.L.); 3 Riga East University Hospital, Clinic of Chemotherapy and Hematology, Riga LV-1038, Latvia; marina.soloveicika@aslimnica.lv

**Keywords:** CCR1, CCR2, CLL, CD38, CD19^+^CD5^+^ lymphocytes

## Abstract

Chemokines and their receptors direct migration and infiltration of immune cells. CCR1 and CCR2 maintain sequence similarity and respond to a number of the same chemokines secreted in lymphoid organs. Expression of CD38 on leukemic cells has been associated with poor clinical outcomes in patients with chronic lymphocytic leukemia (CLL) and is considered as the negative predictor of progression. In our study of newly diagnosed CLL patients, which included 39 CD38-positive and 22 CD38-negative patients, CCR1 and/or CCR2 were always detected, using flow cytometry, on the peripheral blood (PB) CD19^+^CD5^+^ lymphocytes in patients with >30% of the CD38^+^ CD19^+^CD5^+^ lymphocytes (*n* = 16). Spearman’s rank correlation analysis determined correlations between the frequency of the CCR1- and CCR2-expressing PB CD19^+^CD5^+^ lymphocytes and the frequency of the CD38-positive CD19^+^CD5^+^ lymphocytes (r_s_ = 0.50 and r_s_ = 0.38, respectively). No significant correlations were observed between *ZAP70* mRNA expression levels in PB mononuclear cells and the frequency of the circulating CCR1^+^ or CCR2^+^ CD19^+^CD5^+^ lymphocytes. Further association studies are needed to verify prognostic relevance of the CCR1/CCR2 expression on leukemic cells in CLL patients at diagnosis. We suggest that CCR1/CCR2 signaling pathways could represent attractive targets for development of CLL anti-progression therapeutics.

## 1. Introduction

Chemokines and their receptors represent an important network of the immune system. Chemokines transmit signals via corresponding cell-surface G protein-coupled receptors. The chemokine receptors CCR1 and CCR2, comprising significant protein sequence homology, share responses to a number of inflammatory chemokines (reviewed in [1,2]) that are induced upon inflammation and under many pathophysiological conditions. Disease association studies, based on animal models and ex vivo human tissues and cells, implicated T-cells and monocyte/macrophage expression of CCR1 and/or CCR2 in the development of inflammatory disorders, autoimmune diseases, and cancer progression (reviewed in [3]).

CCR2 expression on human B lymphocytes in peripheral blood (PB) and tonsils was first reported in 1997 by Frade et al. [4]. Both CCR1 and CCR2 were detected on B cells isolated from tonsils of healthy donors, on memory (CD38^−^ IgD^−^) and naïve (IgD^+^) non-germinal center B cells, but not on germinal center (GC) B cells (CD38^+^ IgD^−^) [5]. However, only a small proportion of the PB-circulating normal B lymphocytes expressed CCR2 (2.9–7.9% of Ig^+^ cells), with no difference observed in expression between IgA^+^ and IgG^+^ cells [6]. The CD19^+^/HLA-DR^+^ B cells in the PB of healthy individuals were also defined by multiparameter flow cytometry analysis as CCR2-negative [7]. Nevertheless, on malignant B cells obtained from 38 patients with non-Hodgkin lymphomas, the cell-surface expression of CCR1 and CCR2 varied in different types of B-cell lymphomas and was detected in 69% and 46%, respectively, of patients with chronic lymphocytic leukemia (CLL), but only in 0% and 17% cases, respectively, of small lymphocytic leukemia [8]. An immunohistochemical study, using hierarchical cluster analysis, demonstrated that CCR1-staining correlated with an activated B-cell type of diffuse large B-cell lymphoma [9]. Previously, we also revealed that both CCR1 and CCR2 are up-regulated in B cells that were isolated from the PB of healthy donors and activated by the in vitro treatment with mitogens (IL-4 and anti-CD40) or upon infection with Epstein–Barr Virus (EBV). Notably, the established lymphoblastoid B-cell lines (LCL) were CD10-negative and expressed CCR1, CCR2, and CD38 at moderate level [10].

Chronic lymphocytic leukemia (CLL) is the most common form of leukemia in adults. It accounts for about 1/4 of the new cases of leukemia. CLL disease is characterized by highly variable clinical courses (reviewed in [11,12]. About 30% of CLL patients survive to more than 15 years [13], including patients who do not require therapy for many years (up to 20 years), and may never need any treatment; while patients with an aggressive form survive no more than 3 years after the diagnosis and require therapy immediately after the diagnosis [11,14,15]. Patients with slowly progressing CLL are not recommended for therapy unless they have evidence of disease progression [12,15]. Chemotherapy-based regimens may cause complications: an increased risk of infections and myelosuppression, and, in a subset of CLL patients, post-therapy myelodysplasia and secondary cancers, such as acute myeloid leukemia (~5%), Richter syndrome (2–7%)—the transformation of CLL to an aggressive lymphoma (DLBCL, Hodgkin lymphoma) (reviewed in [11]). Two clinical staging systems, the Rai and the Binet classification, are widely used in clinical practice to define patients with the early- (low-risk), intermediate-, and late-stage (high-risk) CLL [16,17]. The late-stage, or high-risk, disease is characterized by the presence of pronounced anemia or thrombocytopenia. In daily clinical practice, the indication for the patient treatment depends on the clinical stage and disease-related symptoms [12]. However, traditional staging systems cannot predict the clinical course in CLL patients with the early (low-risk) stage of disease [18,19]. Nowadays, two main types of CLL, namely slowly progressing and rapidly progressing, are distinguished by whether leukemic cells express a mutated or an unmutated immunoglobulin heavy chain variable region (*IGHV*) gene. CLL patients with the unmutated *IGHV* gene in leukemic cells generally have a more aggressive disease than patients with the mutated *IGHV* gene [20,21]. The *IGHV* mutational status is a parameter that is currently determining the choice of therapy [11]. Since CD38 (cyclic ADP-ribose hydrolase 1) expression in CLL cells has been associated with unmutated *IGHV* and shorter overall survival in patients with CLL, CD38 was proposed as a surrogate marker of the *IGHV* somatic mutation status in CLL [20,22,23]. An association between the CD38 expression on PB CLL cells and the more aggressive CLL disease, when patients had the reduced time-to-first treatment, progression-free survival, and overall survival, was confirmed by numerous reports. CD38 is accepted as an indicator of activated CLL cells; CD38-expressing leukemic cells are characterized by enhanced response to B-cell receptor (BCR) signaling and increased cell migration ability (reviewed [24]). Although CD38-expression levels vary throughout the course of the disease [25], CD38 showed better concordance with the *IGHV* mutation status than did tyrosine-protein kinase ZAP-70 (zeta chain of T cell receptor associated protein kinase 70) [26,27].

Three studies reported in 2015 that the high *EBV* DNA load in peripheral blood mononuclear cells (PBMC) (>1000 copies/µg DNA) at CLL diagnosis was significantly associated with therapy response [28], shorter time to disease progression and time to first treatment [29], and a 3.14-fold increased hazard ratio of death and poor overall survival [30].

We focused our study on newly diagnosed patients with the CD38-positive CLL. In this study of 61 newly diagnosed CLL patients, which included 39 patients presenting with CD38 on leukemic cells, we assessed the cell-surface expression of the chemokine receptors CCR1 and CCR2 in the PBMC populations that included CD19^+^CD5^+^, CD19^+^CD5^−^, and CD19^™^ (designated as T-NK) lymphocytes and monocytes, using the multiparameter flow cytometry (mFC) method. We estimated correlations and evaluated the data in relation to expression of the negative prognostic marker CD38 on the CD19^+^CD5^+^ lymphocytes. The *ZAP70* mRNA expression levels in PBMCs and the EBV copy numbers were determined as well.

## 2. Experimental Section

### 2.1. Patients

Sixty-one patients, newly diagnosed with CLL in the Clinic of Chemotherapy and Hematology (CCH) at Riga East University Hospital (REUH, Latvia) during 2014–2019, were included in the study. PB samples were collected from patients who had an increase of B lymphocytes (>5 × 10^9^/L) in the blood. For the primary diagnosis, PB samples were analyzed on the same day by flow cytometry (FC) for the co-expression of the CD markers CD19, CD20, CD22, CD5, CD23, and CD38. The primary CLL diagnosis was made when cells co-expressed the B-cell marker(s) CD19/CD20 (with or without CD22), CD23, and CD5. The Rai classification was applied to characterize the clinical stages [16]. For this report, we re-considered the clinical stages of the involved CLL patients according to the recommendations of the consensus guidelines of the International Workshop on Chronic Lymphocytic Leukemia (IWCLL), which were updated in 2018 [12]. According to this updated classification, patients with blood B-cell lymphocytosis (>5 × 10^9^/L), the immunophenotype CD19^+^/CD20^+^CD23^+^CD5^+^ of PB cells, and disease-related anemia (blood hemoglobin concentration < 11 g/dL) or thrombocytopenia (platelet blood count < 100 × 10^9^/L) were defined as patients with the high-risk disease (Rai stages III and IV, respectively). Intermediate-risk disease included patients with blood lymphocytosis, the typical immunophenotype, and lymphadenopathy or splenomegaly and/or hepatomegaly (Rai stage I and II, respectively). Patients with the typical immunophenotype and only lymphocytosis were defined as having the low-risk disease (Rai stage 0).

The REUH and Riga Stradins University (RSU, Latvia) Ethics Committees for Medical and Biomedical Research approved the study (ethics approval No 6-A/14 from 05 July 2014 and ethics approval No 6-3/4/19 from 25 April 2019, respectively). Informed consent was obtained from all patients involved in the study.

### 2.2. Epstein–Barr Virus (EBV) DNA Load Quantification

The EBV DNA copy number in the PBMC DNA samples of CLL patients was determined using an EBV Real-TM Quant Kit (Sacace Biotechnologies S.r.l., Como, Italy) with a sensitivity of not less than 200 copies/mL or 5 copies of *EBV* DNA per 10^5^ cells. Real-time polymerase chain reaction (RT-PCR) was performed in duplicate according to the kit protocol in the CFX96 Touch Real-Time PCR Detection System (Bio-Rad Laboratories Inc., Richmond, CA, USA).

### 2.3. Flow Cytometry Analyses

After the primary CLL diagnosis in the clinical FC laboratory, the PB samples were stained with fluorochrome-conjugated anti-human mouse monoclonal antibodies (mAbs) according to the protocol, “Direct Immunofluorescence Staining of Whole Blood Using a Lyse/Wash Procedure” (BD Biosciences, San Jose, CA, USA). The cell-surface expression of CD45, CD19, CD5, CD14, CD38, CD191 (CCR1), CD192 (CCR2), and the membrane phospholipid phosphatidylserine (PS) in the PBMC populations was assessed by seven-color mFC. The following fluorochrome-conjugated mAbs, purchased from BD Biosciences, were used: CD45-Alexa Fluor 700 (clone HI30), CD19-FITC (clone HIB19), CD5-PE-Cy7 (clone L17F12) or CD5-BV510 (clone UCHT2), CD38-Horizon V450 (clone HB7), CD14-PE (clon MφP9), CD191-Alexa Fluor 647 (clone 53504), and CD192-Alexa Fluor 647 (clone 48607). Annexin V-PerCP-Cy5.5 and nonspecific mouse IgG1 and IgG2b, matching the respective fluorochrome and Ig-isotype of CD191 and CD192, were applied as the controls. For the analysis of the cell-surface expression of CCR1 (NP_001286) and CCR2 (NP_001116868/NP_001116513), we used mouse mAbs CD191-Alexa Fluor 647 (clone 53504) and CD192-Alexa Fluor 647 (clone 48607) in our previous work, where we confirmed the CCR1 and CCR2 expression on the cell-surface of B cells in Burkitt lymphoma and lymphoblastoid cell lines, defined using flow cytometry, by the fluorescent immunostaining, Western blot, RT-PCR, and cell migration analyses [10]. Apoptotic cells, which express the membrane PS, were stained with Annexin V-PerCP-Cy5.5 and were excluded from the analyses [31]. Autofluorescent cells were removed from each analysis by gating on viable cells in 2 ways, namely by using the PerCP-Cy5.5 fluorescence channel and by using the SSC/FSC dot-plots of the unstained cells. The CD45^+^ leukocytes were divided into granulocyte, monocyte (CD14^+^), and lymphocyte populations by gating on the SSC/FSC and SSC/PE dot-plots. The CD19-negative lymphocytes were counted and designated as T-NK lymphocytes. Seven-color mFC was performed; cells were scored and analyzed using the BD FACSAriaII or FACSAriaIIIu analyzers and Diva8.2 software (Becton, Dickinson and Company, Franklin Lakes, NJ, USA).

### 2.4. Statistical Analyses

Percentages of the CCR1- and/or CCR2-positive cells within the PBMC populations were summarized and presented as the medians with interquartile ranges (IQR: 25% percentile and 75% percentile). Data of clinical parameters, in the cases of unequal standard deviations, were presented as the medians and interquartile ranges. Data that were not normally distributed were analyzed using the Kruskal–Wallis (KW) test followed by post-hoc analysis, the two-stage step-up method of Benjamin, Krieger and Yekutieli (BKY). To compare proportions and differences between patient groups, the Chi-square test (Chi^2^) was performed. The Spearman’s rank correlation analysis was applied to determine the associations between the frequencies of the CCR1-, CCR2-, and CD38-positive cells within the PB CD19^+^CD5^+^ lymphocytes and the clinical parameters. Determined Spearman’s rank correlation coefficients (r_s_) were reported as the correlation matrix images. The level of significant difference was selected using two-sided p-values less than 0.05 (*p* < 0.05). Statistical analyses were done using the GraphPad Prism version 8.0 (GraphPad Software, San Diego, CA, USA).

### 2.5. Real-Time Polymerase Chain Reaction (RT-PCR) Analysis of ZAP70 Expression

The *ZAP70* (zeta chain of T cell receptor associated protein kinase 70; RefSeqGene NG_007727) gene expression was assessed in whole PBMC fractions at the mRNA level via real-time RT-PCR analysis. Real-time RT-PCR was performed using the first-strand cDNA (prepared from the total RNA that was reverse-transcribed with the random hexamer primer), gene-specific primers, the PerfeCTa SYBR Green FastMix (Quanta BioSciences Inc., Beverly, MA, USA), and the CFX96 Touch Real-Time PCR Detection System (Bio-Rad Laboratories Inc., Richmond, CA, USA). The *ZAP70*-specific primers and PCR protocol were described previously by Gladkikh et al. [32]. The values were normalized to the mRNA expression levels of *GUSB* (glucuronidase beta). This gene has been reported to be one of the three most suitable reference genes for PCR studies of CLL PB B cells [33]. A control cDNA sample, prepared from PBMC of the CLL patient with the low-moderate level of *ZAP70* mRNA expression, was applied in each PCR run as an inter-run calibrator (IRC). The data were calculated relative to the IRC levels.

## 3. Results

### 3.1. The Patient Groups

For this study, we re-considered the Rai clinical stages [16] of the involved CLL patients according to the recommendations of the consensus guidelines of the International Workshop on Chronic Lymphocytic Leukemia (IWCLL), which were updated in 2018 [12]. The newly diagnosed CLL patients (*n* = 61) were divided into three groups depending on the frequency of the CD38-positive cells within the PB CD19^+^CD5^+^ lymphocytes. The CD38-negative group (CD38^−^ group) included patients (*n* = 22) with a frequency of the CD38^+^CD19^+^CD5^+^ cells less than 6.0%. In the CD38-moderate group (CD38^+^ group) (*n* = 23), the percentages of the CD38^+^CD19^+^CD5^+^ cells ranged from 6.0% to 30.0% (median: 19.8%). Finally, the CD38-positive group (CD38^++^ group) included patients (*n* = 16) with more than 30% (median: 54.6%; range: 33.4–79.3%) of the CD38^+^ cells within the circulating CD19^+^CD5^+^ lymphocytes. The clinical parameters of the newly diagnosed CLL patients included in the cohort groups are shown in Table 1.

**Figure 1:**
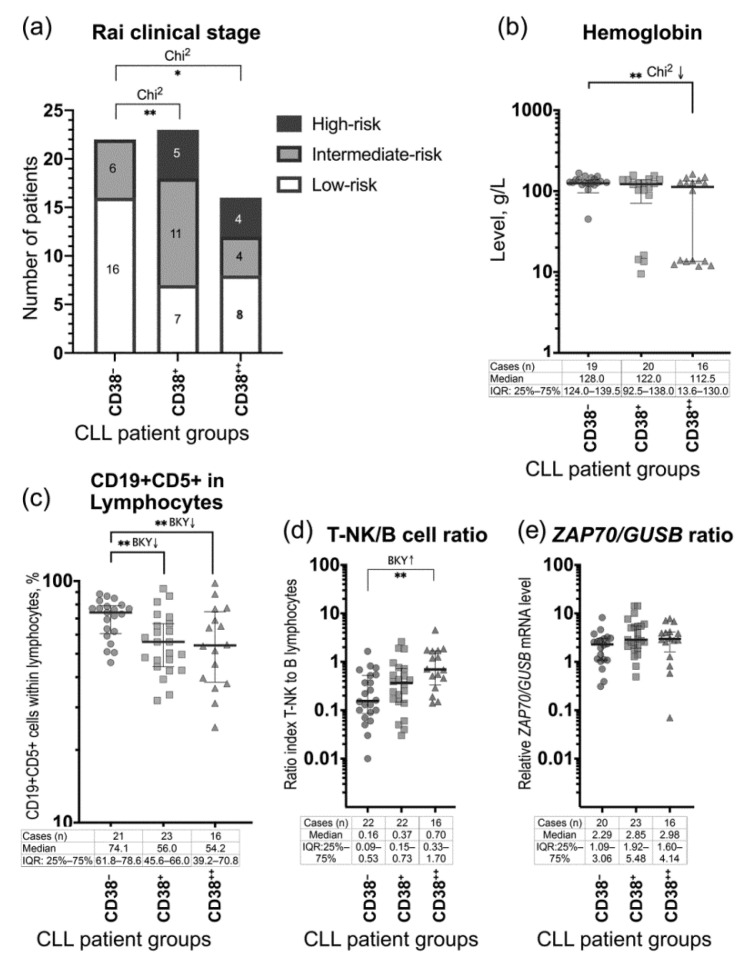
Statistically significant clinical parameters of the newly diagnosed CD38-negative and CD38-positive chronic lymphocytic leukemia (CLL) patients. (**a**) The frequency of the clinical stages by Rai classification within the CD38^−^ group and the CD38-positive groups, the CD38^+^ group and the CD38^++^ group. (**b**) The peripheral blood (PB) hemoglobin levels in the CD38-negative and CD38-positive groups of patients. (**c**) The percentage of the CD19^+^CD5^+^ cells within the lymphocyte set in the CD38^−^ group and the CD38-positive groups. (**d**) T-NK/B cell ratios, the ratios of the frequency of CD19^−^ lymphocytes (designated as T-NK lymphocytes) to the frequency of CD19^+^ B lymphocytes within the PBMC set of the patients were determined using flow cytrommetry. (**e**) The relative *ZAP70* mRNA expression levels in the peripheral blood mononuclear cells (PBMC) of CLL patients was determined using real-time polymerase chain reaction (RT-PCR); the *ZAP70* mRNA expression values were normalized to the housekeeping gene *GUSB* mRNA expression values (*ZAP70/GUSB*) and the levels of *ZAP70* mRNA expression were calculated relative to the inter-run calibrator. CD38^−^, the CD38^−^ group of patients with <6.0% of the CD38^+^ cells within the PB CD19^+^CD5^+^ lymphocytes; CD38^+^, the CD38^+^ group of patients with 6.0‒30.0% of the CD38^+^ cells within the PB CD19^+^CD5^+^ lymphocytes; CD38^++^, the CD38^++^ group of patients with >30.0% of the CD38^+^ cells within the PB CD19^+^CD5^+^ lymphocytes. The median and IQR (25% percentile and 75% percentile) values are presented in the tables; BKY, the Benjamin, Krieger and Yekutieli test; ** *p* < 0.01.

The clinical Rai staging classification showed the concordance with the proportions of the CD38-expressing CD19^+^CD5^+^ lymphocytes (Table 1). In the CD38^−^ group, there were no patients with the high-risk disease (the Rai stages III–IV), and the majority of patients (72.7%) had the low-risk disease (Rai stage 0) at the time of diagnosis. By contrast, in the CD38^++^ group, 25% of patients had the intermediate-risk disease (the Rai stages I-II) and 25% of patients were with the high-risk disease (the Rai stages III-IV). Noteworthy, the frequency of the Rai clinical stages differed statistically significant between the CD38-negative group and the CD38-positive groups, namely the CD38^+^ group and the CD38^++^ group, but not between two CD38-positive groups (Figure 1a). We observed the notable prevalence of male patients and lower ages in the CD38^++^ group. The majority of the clinical parameters did not differ significantly between the groups. However, the percentage of the CD19^+^CD5^+^ cells within the lymphocyte set differed statistically significant between the CD38-negative group and the CD38-positive groups (the CD38^+^ group and the CD38^++^ group) of the newly diagnosed patients (Figure 1c). Significant difference of the hemoglobin levels was observed between the CD38^−^ group and the CD38^++^ group of patients (Table 1, Figure 1b). Evidently, these results require validation in a large CLL patient cohort.

In our study of 61 newly diagnosed CLL patients, 58 patients had fewer than 50 copies of *EBV* DNA per 1 µg of PBMC DNA (or <30 *EBV* DNA copies per 10^5^ PBMCs). More than 400 copies of *EBV* DNA per 1 µg were found in three patients only. Two patients from the CD38^+^ group had ~441 copies/µg (with the frequency of the CD38^+^, CCR1^+^, and CCR2^+^ cells within the CD19^+^CD5^+^ lymphocytes of 10.1%, 10.6%, and 13.0%, respectively) and ~871 copies/µg (with the frequency of the CD38^+^, CCR1^+^, and CCR2^+^ cells within the CD19^+^CD5^+^ lymphocytes of 29.6%, 28.4%, and 22.7%, respectively) *EBV* DNA copies, respectively, and one patient from the CD38^++^ group had ~430 copies/µg of the *EBV* DNA copies (with the frequency of the CD38^+^, CCR1^+^, and CCR2^+^ cells within the CD19^+^CD5^+^ lymphocytes of 53.0%, 26.8%, and 26.3%, respectively).

### 3.2. Expression of CCR1 and CCR2 on Peripheral Blood Mononuclear Cells

**Figure 2:**
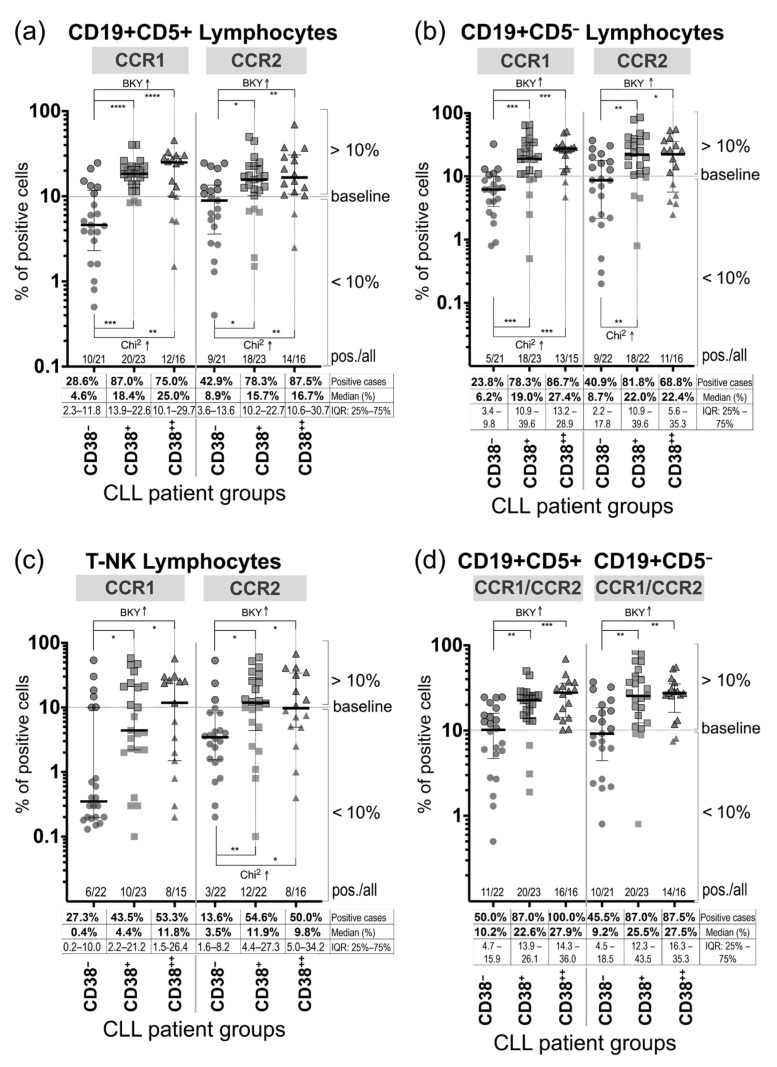
Frequency of the CCR1- and CCR2-expressing peripheral blood lymphocytes in the newly diagnosed CD38-negative and CD38-positive CLL patients. Frequency of the CCR1^+^ and CCR2^+^ cells within the CD19^+^CD5^+^ lymphocytes (**a**), CD19^+^CD5^−^ lymphocytes (**b**), and CD19^−^ lymphocytes (designated as T-NK lymphocytes) (**c**) are shown. (**d**) Frequency of the CCR1^+^ or CCR2^+^ (designated as CCR1^+^/CCR2^+^) CD19^+^CD5^+^ lymphocytes and CD19^+^CD5^−^ lymphocytes. CD38^−^, the CD38^−^ group of patients with <6.0% of the CD38^+^ cells within the PB CD19^+^CD5^+^ lymphocytes; CD38^+^, the CD38^+^ group of patients with 6.0‒30.0% of the CD38^+^ cells within the PB CD19^+^CD5^+^ lymphocytes; CD38^++^, the CD38^++^ group of patients with >30.0% of the CD38^+^ cells within the PB CD19^+^CD5^+^ lymphocytes. For CCR1 and CCR2, ≥10.0% of the fluorochrome-stained cells within the selected gate were considered to be positive cells; the positivity threshold border (baseline) of 10.0% was defined in our study for the CD191/CD192–Alexa 647 stained cells, based on the fluorochrome staining recorded for the control Ig-isotype antibodies and Annexin V–PerCP-Cy5.5. The percentages of positive cells from two experiments (with two tubes per experiment) were averaged. Data were collected when at least 100 cells displayed the immunophenotype. On plots, the median and IQR (25% percentile and 75% percentile) values are shown; Chi^2^, the Chi–square test; BKY, the method of Benjamin, Krieger and Yekutieli; * *p* < 0.05, ** *p* < 0.01, *** *p* < 0.001, **** *p* < 0.0001.

Expression of CCR1 and CCR2 on the cell surface of the PB lymphocytes, CD19^+^CD5^+^, CD19^+^CD5^−^, and CD19^−^ (designated as T-NK lymphocytes), and PB monocytes was determined in CLL patients in all groups. Nevertheless, differences in the number of the CCR1- and CCR2-positive patients and the frequency of the CCR1^+^ and CCR2^+^ PB lymphocytes were statistically significant between the CD38^−^ group and the CD38-positive groups, the CD38^+^ group and the CD38^++^ group (Figure 2a–c). Notably, in the CD38^−^ group, the percentage median values of the CCR1^+^ and CCR2^+^ lymphocytes, i.e., CD19^+^CD5^+^, CD19^+^CD5^−^, and T-NK lymphocytes, were below the positivity threshold border (baseline) of 10.0%.

The CCR1^+^ and CCR2^+^ CD19^−^ lymphocytes (designated as T-NK lymphocytes) were also found in patients in all groups, but the percentage median values of the positive cells were located below or, in two instances, close to the positivity baseline (10.0%), namely 11.8% of the CCR1^+^ T-NK lymphocytes in the CD38^++^ group and 11.9% of the CCR2^+^ T-NK lymphocytes in the CD38^+^ group (Figure 2c). In the CD38^++^ group, only 53.3% of patients were CCR1-positive and 50.0% of patients were CCR2-positive, with ≥10.0% (the threshold border) of the positive T-NK lymphocytes, respectively.

Notably, the number of patients with the CCR1^+^ and CCR2^+^ CD19^+^CD5^+^ lymphocytes was essentially higher in the CD38-positive groups compared to the CD38^−^ group: 87.0% and 78.3%, respectively, of the cases in the CD38^+^ group, and 75.0% and 87.5%, respectively, of the cases in the CD38^++^ group versus 28.6% and 42.9%, respectively, in the CD38^−^ group (Figure 2a). The differences in frequency of the CCR1- and CCR2-positive CD19^+^CD5^+^ lymphocytes were statistically significant between the CD38^−^ group and the CD38-positive groups and the percentage median values were also higher in the CD38-positive groups than in the CD38^−^ group: 18.4% and 15.7% in the CD38^+^ group, respectively, and 25.0% and 16.7% in the CD38^++^ group, respectively, versus 4.6% and 8.9% (below the positivity baseline of 10.0%), respectively, in the CD38^−^ group.

The higher number of patients with the CCR1^+^ and CCR2^+^ CD19^+^CD5^−^ lymphocytes and a higher frequency of the CCR1^+^ and CCR2^+^ CD19^+^CD5^−^ lymphocytes were also observed in the CD38-positive groups relative to the CD38^−^ group (Figure 2b): 78.3% of the CCR1^+^ cases with the percentage median value of 19.0%, and 81.8% of the CCR2^+^ cases with the median value of 22.0% in the CD38^+^ group, and 86.7% of the CCR1^+^ cases with the median value of 27.4%, and 68.8% of the CCR2^+^ cases with the median value of 22.4% in the CD38^++^ group versus 23.8% of the CCR1^+^ cases (the median value: 6.2%) and 40.9% of the CCR2^+^ cases (the median value: 8.7%) in the CD38^−^ group. The differences between the CD38^−^ group and the CD38-positive groups were statistically significant (Figure 2b).

Both CCR1 and CCR2 respond to a number of the same chemokines and can duplicate a function or substitute each other [1,2,34]. Therefore, we estimated the frequency of the PB lymphocytes (CD19^+^CD5^+^, CD19^+^CD5^−^, and T-NK) that were expressing one receptor or another (designated as CCR1^+^/CCR2^+^) (Figure 2d). The number of patients with the CCR1^+^/CCR2^+^ CD19^+^CD5^+^ lymphocytes was 1.7-fold higher in the CD38^+^ group (87.0%) and 2.0-fold higher in the CD38^++^ group (100%) compared to the CD38^−^ group (50.0%). The differences in the frequency median values of the CCR1^+^/CCR2^+^ CD19^+^CD5^+^ lymphocytes were statistically significant between the CD38^−^ group and the CD38-positive groups, namely the CD38^+^ group and the CD38^++^ group, and the median values were also 2.2- and 2.7-fold higher in the CD38^+^ group and the CD38^++^ group, respectively, than in the CD38^−^ group (Figure 2d). We also determined that in the CD38-positive groups, there was a higher number of patients with the CCR1^+^/CCR2^+^ CD19^+^CD5^−^ lymphocytes; a 1.9-fold increase was observed in the CD38^+^ group (87.0% of the positive cases) and in the CD38^++^ group (87.5% of the positive cases), relative to the CD38^−^ group (45.5% of the positive cases). The frequencies of the CCR1^+^/CCR2^+^ CD19^+^CD5^−^ lymphocytes were 2.9- and 3.1-fold higher in the CD38-positive groups, the CD38^+^ group and the CD38^++^ group, respectively, than in the CD38^−^ group and these differences were also significant (*p* < 0.01) (Figure 2d).

**Figure 3:**
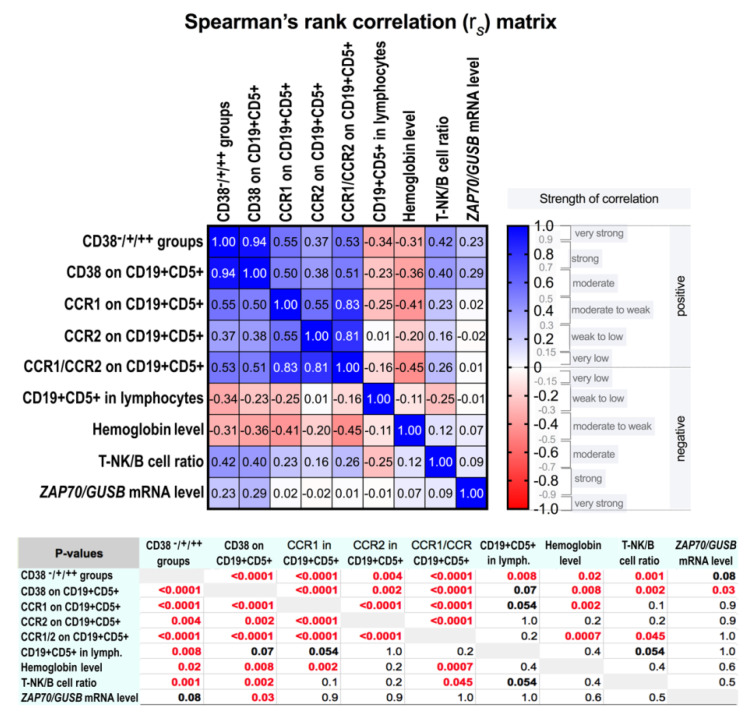
Spearman’s rank correlation analysis of the frequencies of the CD38-, CCR1-, and CCR2-expressing peripheral blood (PB) CD19^+^CD5^+^ lymphocytes in the newly diagnosed CLL patients. The Spearman’s rank correlation analysis matrix is shown. CD38 on CD19^+^CD5^+^, the frequency of the CD38-expressing cells within the PB CD19^+^CD5^+^ lymphocytes; CCR1 on CD19^+^CD5^+^ and CCR2 on CD19^+^CD5^+^, the frequencies of the corresponding receptor-expressing cells within the PB CD19^+^CD5^+^ lymphocytes; CCR1/CCR2 on CD19^+^CD5^+^, the frequency of the CCR1- or CCR2-expressing cells within the PB CD19^+^CD5^+^ lymphocytes; *ZAP70/GUSB*, the relative normalized-to-*GUSB ZAP70* mRNA expression levels in the PBMC fractions; T-NK/B cell ratio, the ratios of the frequency of CD19^−^ lymphocytes (designated as T-NK lymphocytes) to the frequency of CD19^+^ B lymphocytes. In the table, the corresponding P-values are presented. Note, the p-values between the *ZAP70* mRNA expression levels and the frequency of CCR1, CCR2, and CCR1/CCR2 on the CD19^+^CD5^+^ lymphocytes are exceeding the significance cut-off value of 0.05.

The overall Spearman’s rank correlation analysis of all the CLL patients in the study revealed positive correlations between expression of CD38 on the PB CD19^+^CD5^+^ lymphocytes and the presence of CCR1, CCR2, and CCR1/CCR2 on these lymphocytes. The Spearman’s rank correlation coefficients were defined as follows: r_s_ = 0.50 (*p* < 0.0001), r_s_ = 0.38 (*p* = 0.002), and r_s_ = 0.51 (*p* < 0.0001), respectively (Figure 3). It should be noted that only a part of the CD38^+^CD19^+^CD5^+^ cells expressed CCR1 or CCR2. Interestingly, the blood hemoglobin levels correlated negatively with the frequency of the CD38^+^ and CCR^+^/CCR2^+^ CD19^+^CD5^+^ PB lymphocytes (r_s_ = −0.36, *p* = 0.01 and r_s_ = −0.45, *p* = 0.0007, respectively) (Figure S1). The other clinical parameters used in the study (Table 1) did not correlate with the CD38- or CCR1-/CCR2-positivity of the CD19^+^CD5^+^ PB lymphocytes. Although, the correlations were observed between the lymphocyte phenotype markers, CD19, CD23, and CD3, and the blood cell counts of the white blood cells (WBC), red blood cells (RBC), neutrophils, lymphocytes, and monocytes (Figure S1).

Expression of CCR1 and CCR2 on the PB monocytes was detected in all the study patients. The percentage median values of the CCR1- and CCR2-expressing monocytes exceeded 50.0% in all three groups, with no statistically significant differences between the groups. The values were as follows: 50.3% (IQR: 35.2–61.0%) and 66.5% (IQR: 40.5–75.9%), respectively, in the CD38^−^ group; 51.3% (IQR: 35.3–63.8%) and 72.1% (IQR: 53.7–83.3%), respectively, in the CD38^+^ group; and 65.0% (IQR: 41.7–82.3%) and 69.0% (IQR: 47.5–84.1%), respectively, in the CD38^++^ group.

### 3.3. ZAP70 Relative mRNA Expression in the Peripheral Blood Mononuclear Cells

We determined the relative normalized-to-*GUSB ZAP70* mRNA expression levels in the PBMC fractions of the patients. Proportions of the CD19^−^ lymphocytes (designated as T-NK) and CD19^+^ lymphocytes (B) within the PBMC set were assessed, using mFC, and the ratios of the T-NK to B lymphocytes (T-NK/B) were calculated. In the CD38^−^ group, the relative *ZAP70*-expression level median value was 2.29 (IQR: 1.09–3.06) (Figure 1e). This median value was taken as the baseline for comparison of the relative *ZAP70* mRNA expression levels in the CD38-positive patients. In our study, the ≥3-fold elevated *ZAP70* expression levels, compared to the median value of 2.29 (baseline) in the CD38^−^ group, were only found in four patients (17.4%) in the CD38^+^ group, namely 7.61 (with T-NK/B = 0.14), 10.03 (with T-NK/B = 0.43), 14.22 (with T-NK/B = 0.06), and 14.25 (with T-NK/B = 0.24); and in three patients (18.8%) in the CD38^++^ group, namely 6.81 (with T-NK/B = 1.25), 7.09 (with T-NK/B = 0.71), and 7.79 (with T-NK/B = 0.55). The median values in the CD38^+^ group and CD38^++^ group were as follows: 2.85 (range: 0.49–14.25) and 2.98 (range: 0.07–7.79), respectively, for the relative *ZAP70* expression levels, and 0.37 (range: 0.03–2.59) and 0.70 (range: 0.14–4.52), respectively, for the T-NK/B ratios (Figure 1e,d). The PBMC relative *ZAP70* mRNA expression levels had no statistically significant differences between three groups of the study CLL patients. However, the PBMC *ZAP70* mRNA expression levels correlated weak-to-low positively with the presence of CD38 on the CD19^+^CD5^+^ lymphocytes (r_s_ = 0.29; *p* = 0.03) (Figure 3). Nevertheless, no correlations were detected between the relative *ZAP70* expression levels and the T-NK/B ratios, as well as the frequency of CCR1, or CCR2 on the CD19^+^CD5^+^ lymphocytes (Figure 3). Notably, while the T-NK/B ratios correlated with the frequency of CD38 on the CD19^+^CD5^+^ lymphocytes, the clinical parameter “proportions of CD19^+^CD5^+^ cells within lymphocytes” did not (Figure 3).

## 4. Discussion

Within our cohort of newly diagnosed CLL patients (*n* = 61), which included 39 patients with the CD38-expressing leukemic cells, we determined a positive correlation between expression of CD38 (the negative predictor of CLL progression) and expression of the chemokine receptors CCR1 and CCR2 on the PB CD19^+^CD5^+^ lymphocytes (r_s_ = 0.50 and r_s_ = 0.38, respectively). Noteworthy, in the all patients with ≥30% of the CD38^+^ CD19^+^CD5^+^ lymphocytes (the CD38^++^ group, *n* = 16), we determined CCR1 or CCR2 or both on the CD19^+^CD5^+^ cells (with the median cumulative frequency of 27.9%). In 87.0% of patients in the CD38^+^ group (with ≥6% and <30% of the CD38^+^ CD19^+^CD5^+^ lymphocytes, *n* = 23), these receptors were also detected on the CD19^+^CD5^+^ cells (with the median cumulative frequency of 22.6%). While in the CD38^−^ group (*n* = 22), CCR1 or CCR2 were found in 50.0% of the patients, the frequency median value of 10.2% was near the positivity cut-of border of 10.0%. Interestingly, the CCR1 and CCR2 expression on the PB CD19^+^CD5^−^ and CD19^−^ (designated as T-NK) lymphocytes was also enhanced in the CD38-positive patients, and the differences between the CD38-negative group and the CD38-positive groups were significant. Extrinsic signals of the microenvironment can be involved into interplay between PB cell subsets. In the case of the cell-surface receptors, as chemokine receptors, the lack of the ligands (antagonists or agonists) allow a receptor to escape internalization into the cell cytoplasm for the further processing and recycling [2]. The relevance of the CCR1 and CCR2 expression in distinct PB cell and lymphocyte subsets to the CLL pathogenesis is required to be investigated.

CCR1 and CCR2 in monocytes, macrophages, and T cells have been studied in many diseases (reviewed in [1,3]). However, the function of CCR1 or CCR2 in B lymphocytes is currently unknown. Earlier, we reported the up-regulation of CCR1 and CCR2 in PB B cells activated in vitro [10] and in circulating B and T lymphocytes in patients with rheumatoid arthritis [35]. CCR1 and CCR2 comprise protein sequence similarity [2] and respond to a number of the same chemokines, which are abundantly secreted in the lymphoid organs (reviewed in [1,3,34,36]). Both CCR1 and CCR2 respond to the chemokines CCL7, CCL8, and CCL16, which are expressed at high levels in the tissues of bone marrow (CCL7), spleen (CCL8), tonsil (CCL8), and liver (CCL16). Besides, the CCR1 ligands (endogenous agonists) are abundantly secreted in bone marrow (CCL3), lymph nodes (CCL4, CCL14), spleen (CCL3, CCL14), and liver (CCL14, CCL15). The CCR2-ligand CCL2 is found in the liver tissues [34,36]. In our earlier studies, we demonstrated the enhanced migration of the CCR2-expressing B cells toward the CCR2 dominant ligand chemokine CCL2 (MCP1) [10,37]. Chemotactic migration of the CCR1/CCR2-expressing CLL cells into the secondary organs, such as lymph nodes, spleen, and/or liver, contributes, apparently, to the aggressive pathogenesis of the disease.

In two recent reports, a mechanism of action for homing chemokine receptors, CCR7, CXCR3, CXCR4, and CCR2, in CLL cells via p66Shc, an adaptor protein, was suggested [38,39]. In B lymphocytes, p66Shc acts as a negative regulator of the BCR-activated signaling and the chemotactic responses, but promotes lymphocyte apoptosis. Since the p66Shc expression was impaired in CLL cells compared to the healthy subjects, involvement of this adaptor protein in CLL pathogenesis was implied. Noteworthy that expression of the CCR7, CXCR3, and CCR2 on CLL cells negatively correlated with the p66Shc expression [38]. The authors described the putative mechanisms whereby p66Shc deficiency in CLL cells induces expression of the homing chemokine receptors and down-regulates expression of the egress receptor S1PR1 (reviewed in [39]).

Higher EBV DNA-copy number (>1000 copies/µg of DNA) in PBMCs of patients at CLL diagnosis was significantly associated with other established unfavorable prognostic factors, such as the *IGHV* mutational status, CD38-positive or ZAP-70-positive phenotype of CLL cells, and the presence of the del(11q22.3) cytogenetic abnormality [28,29]. On the contrary, the multivariate analysis showed that the poor prognosis of the high EBV DNA load was reflecting more prominent tumor aggressiveness, regardless of clinical stage of the CLL disease, or age. Furthermore, no significant relationship between EBV DNA-copy number and unmutated *IGHV* was observed. Thus, the multivariate analysis demonstrated that the EBV DNA load at diagnosis is an independent predictor of overall survival in patients with CLL [30]. A quantitative test for EBV DNA load was suggested to be integrated in the assessment of patients with newly diagnosed CLL to evaluate their outcome [28,29,30]. However, in our study of 61 newly diagnosed CLL patients, more than 50 copies of *EBV* DNA per 1 µg of PBMC DNA were determined in 3 patients only, reflecting, possibly, the geographic and/or seasonal impact on EBV activation and transient immunosuppression.

Next-generation sequencing studies have disclosed intra-tumor heterogeneity in CLL. Some somatic mutations (as in MYD88) and chromosomal abnormalities (as del(13q) and trisomy 12) are mostly found in the all leukemic cells indicating that these alterations occurred early in leukemia genesis. Other mutations (as in *SF3B1* and *NOTCH1*) and chromosomal abnormalities (as del(17p) are often found in a fraction of the CLL cells, thus representing sub-clonal diversity, which occurs later in the development of CLL. Sub-clonal distribution of mutations is associated with more aggressive form of the disease (reviewed in [11,12]). Nevertheless, the previous studies reported that the *IGHV* mutational status has prognostic significance independent of cytogenetic findings in CLL [40,41]. Interphase fluorescence in situ hybridization (FISH) and TP53 mutation analyses are not essential to diagnose CLL but help predict the treatment response and should guide therapeutic decisions [12].

Until now, the somatic mutation status of the *IGHV* gene is the most valuable prognostic in CLL [11]. However, identification of the expressed *IGHV* segments and the *IGHV* mutation status, the FISH and mutation analyses are more expensive and complicated than flow cytometry tests for routine clinical practice and are still unavailable to many patients, particularly in Eastern European countries. In Latvia, the government healthcare system financially supports the FISH and mutation analyses only for CLL patients with the age ≤50 years old and patients who did not respond to the first-line therapy. Currently, two indicators of the CLL high-risk progression, ZAP-70 and CD38 in leukemic cells, are being used in routine clinical FC tests. However, ZAP-70 showed worse concordance with the *IGHV* mutation status than did CD38 [26,27].

In the rather small cohort of the untreated CLL patients in our study (*n* = 61), we did not observe any correlation between the *ZAP70* mRNA expression levels in the PBMCs and the CCR1- or CCR2-positivity of the PB CD19^+^CD5^+^ lymphocytes. However, the PBMC *ZAP70* mRNA expression levels correlated positively moderate-to-week (r_s_ = 0.29) with the frequency of the CD38^+^ PB CD19^+^CD5^+^ lymphocytes, according to the Spearman’s rank correlation statistical analysis (Figure 3). In an early study of 107 CLL patients, in 93% of patients the *ZAP70* expression level was 5.54-fold higher in patients with unmutated *IGHV* than in patients with mutated *IGHV*, and thus, ZAP-70 was suggested to be a prognostic indicator in CLL [42]. However, in another study of 127 CLL patients, ZAP-70 protein expression was not predictive of survival in patients with Binet’s stage B and C [43].

CD38 expression on the PB leukemic cells has been reported as a CLL negative prognostic factor, predicting shorter overall survival, in numerous publications, but with various cut-off levels. In the first report in 1999 and the following studies, CD38 expression predicted survival rates when ≥30% of leukemic cells were determined as CD38-positive [25,28,44]. In other studies, the cut-off levels of 20% [45,46] or even 7% [47,48] of the CD38-expresing CLL cells were applied. In our study, we divided the CD38-positive CLL patients into two groups, the CD38^+^ group and the CD38^++^group, with the cut-off levels of 6.0–30.0% and >30.0%, respectively, of the CD38-positive cells within the circulating CD19^+^CD5^+^ lymphocytes. Noteworthy that the Rai clinical stages and the frequency of the CCR1^+^/CCR2^+^ lymphocytes differed statistically significantly between the CD38-negative group and the CD38^+^ group, and between the CD38-negative group and the CD38^++^ group, but not between two CD38-positive groups. Taking into account the correlation analyses data, our results indicate the CD38 expression on >6% of the PB CD19^+^CD5^+^ lymphocytes as a risk factor in CLL patients at diagnosis. Further efforts are necessary to establish the common cut-off level of the CD38-expresing cells for the risk evaluation in CLL.

The strong association of the CD38 and CD49d (the alpha chain of the alpha4beta1 integrin heterodimer) expression on CLL cells has been first reported by Zucchetto and coauthors [49] and then confirmed in several studies. CD49d was shown to be an independent prognostic marker in patients with CLL (reviewed in [50]). The combined CD38/CD49d phenotype was the strongest FC-based predictor of overall survival and the treatment-free survival in large cohorts of patients with CLL [50,51], supporting the previous suggestion of the scoring system based on the expression of several cell-surface antigens for correct risk stratification in CLL [52].

New predictor markers, i.e., reliable prognostic indicators of the high-risk progression in CLL patients at disease presentation, which can be introduced into routine diagnostic procedures (such as the immunophenotypical identification using flow cytometry), are still needed. We demonstrated an association of the CCR1/CCR2 expression on the circulating CD19^+^CD5^+^ lymphocytes with the CD38-positivity of these lymphocytes. Our results suggest that flow cytometric detection of CCR1 and CCR2 on the PB leukemic cells, along with CD38, could serve as an additional indicator predicting the high risk of disease progression in CLL patients at diagnosis.

We are reporting that in newly diagnosed CLL patients with the expression of the high-risk progression marker CD38 on leukemic cells, the chemokine receptors CCR1 and CCR2 were up-regulated on the PB CD19^+^CD5^+^ lymphocytes, indicating their increased migration capability toward chemokine-ligands secreting organs. Functions of the chemokine receptors CCR1 and CCR2 in B lymphocytes, in healthy or pathological conditions, are largely unknown. Disclosure of the role of CCR1 and CCR2 in the pathogenesis of CLL could suggest these receptors and their signaling pathways as targets for the development of anti-progression therapeutics in CLL.

## 5. Conclusions

In our study of 61 newly diagnosed CLL patients, which included 39 patients with CD38-positive leukemic cells, we demonstrated the presence of the chemokine receptors CCR1 and CCR2 on the circulating CD19^+^CD5^+^ and CD19^+^CD5^−^ lymphocytes in the CD38-positive patients. Furthermore, the frequency of the CCR1- and/or CCR2-expressing CD19^+^CD5^+^ lymphocytes positively correlated with the frequency of the CD38-expressing CD19^+^CD5^+^ lymphocytes. Detection of CCR1 and CCR2 on the PB CD19^+^CD5^+^ lymphocytes could be suggested in routine diagnostic FC tests, in addition to CD38, for accurate prognosis of the high-risk progression in CLL patients at diagnosis. However, further association studies with an extended patient cohort are necessary to verify whether the presence of CCR1 and/or CCR2 on circulating leukemic cells is a reliable prognostic indicator in CLL.

## Figures and Tables

**Table 1:** Demographic and clinical parameters of the newly diagnosed chronic lymphocytic leukemia (CLL) patients.

Demographic and Clinical Parameters	CD38^−^ Group ^(1)^ *n* = 22	CD38^+^ Group ^(2)^ *n* = 23	CD38^++^ Group ^(3)^ *n* = 16
Age in years: mean (range)	69.3 (49–86)	70.0 (54–88)	65.6 (41–79)
Gender: Male number (%) Female number (%)	10 (45.5%) 12 (54.5%)	11 (47.8%) 12 (52.2%)	11 (68.8%) 5 (31.2%)
Low-risk disease (Rai stage 0), *n* (%)	16 (72.7%)	7 (30.4%)	8 (50.0%)
Intermediate-risk disease (Rai stages I–II), *n* (%)	6 (27.3%)	11 (47.8%)	4 (25.0%)
High-risk disease (Rai stages III–IV), *n* (%)	0 (0.0%)	5 (21.7%)	4 (25.0%)
Lymphadenopathy, *n* (%)	2 (9.1%)	8 (34.8%)	5 (31.3%)
Splenomegaly, *n* (%)	2 (9.1%)	7 (30.4%)	5 (31.3%)
Leukocytosis (white blood cells), 10^9^/L: Range Median (IQR: Q1–Q3)	8.4–81.8 18.1 (12.6–42.5)	9.3–142.7 18.5 (14.7–65.3)	10.7–133.2 19.3 (15.0–33.3)
Lymphocytosis, 10^9^/L: Range Median (IQR: Q1–Q3)	5.4–66.4 16.6 (7.8–40.8)	2.7–99.0 13.5 (9.4–59.2)	6.7–103.0 14.1 (9.5–32.9)
Monocytosis, 10^9^/L: Range Median (IQR: Q1–Q3)	0.2–8.8 0.6 (0.4–1.9)	0.4–16.1 1.0 (0.5–2.1)	0.4–11.7 0.7 (0.6–1.9)
Neutrophilia, 10^9^/L: Range Median (IQR: Q1–Q3)	1.1–21.9 3.4 (2.9–4.4)	1.9–25.1 4.4 (3.1–6.4)	2.4–10.8 3.7 (3.2–5.2)
Red blood cell count, 10^12^/L: Range Median (IQR: Q1–Q3)	1.1–5.2 4.1 (3.9–4.5)	3.0–5.2 4.3 (3.8–4.6)	3.3–4.8 4.2 (4.1–4.5)
Hemoglobin, g/L: Range Median (IQR: Q1–Q3)	45.0–167.0 128.0 (124.0–139.5)	9.5–157.0 122.0 (92.5–138.0)	11.8–162.0 ** 112.5 (13.6–130.0)
Platelets, 10^9^/L: Range Median (IQR: Q1–Q3)	128.0–285.0 175.0 (147.0–218.0)	57.0–335.0 221.5 (153.8–241.5)	110.0–261.0 173.5 (141.8–206.0)
CD19+ cells (among lymphocytes), %: Range Median (IQR: Q1–Q3)	58.1–97.1 83.7 (76.2–91.1)	47.0–95.9 82.0 (73.5–87.2)	37.3–98.5 78.6 (66.5–85.7)
CD23+ cells (among lymphocytes), %: Range Median (IQR: Q1–Q3)	33.5–85.3 71.6 (59.3–78.6)	10.8–84.6 56.5 (47.5–76.3)	24.7–86.1 60.5 (46.9–71.1)
CD3+ cells (among lymphocytes), %: Range Median (IQR: Q1–Q3)	3.0–23.0 14.5 (7.7–19.8)	3.0–49.0 12.1 (8.5–21.5)	1.1–40.0 13.1 (9.0–22.1)
CD19+CD5+ (among lymphocytes), %: Range Median (IQR: Q1–Q3)	46.0–88.4 74.1 (61.8–78.6)	32.0–93.0 ** 56.0 (45.6–66.0)	24.8–98.0 ** 54.2 (39.2–70.8)
EBV DNA >50 copies/1 ug of PBMC DNA), *n* (copy number)	0/(0.0)	2/(441; 871)	1/(430)
Relative *ZAP70* mRNA level in PBMC: Range Median (IQR: Q1–Q3)	0.31–8.18 2.29 (1.09–3.06)	0.49–14.25 2.85 (1.92–5.48)	0.07–7.79 2.98 (1.60–4.14)

The CD38^−^ group (1) included patients with the frequency of the CD38^+^ cells within the CD19^+^CD5^+^ lymphocytes less than 6.0%; in the CD38^+^ group (2), the percentages of the CD38^+^ CD19^+^CD5^+^ cells ranged from 6.0% to 30.0%; the CD38^++^ group (3)_ included patients with more than 30% of the CD38^+^ cells within the PB CD19^+^CD5^+^ lymphocytes. The threshold border at 6.0% was determined for the CD38-Horizon V450 stained cells based on the fluorochrome staining observed with the control antibodies. Defined statistically significant differences of the parameters are shown between the CD38^−^ group and the CD38^+^ group (in 2) and the CD38^−^ group and the CD38^++^ group (in 3); ** *p* < 0.01. The Chi^2^ test was applied for the hemoglobin levels; the Benjamin, Krieger and Yekutieli (BKY) post-hoc analysis was performed for the percentage values of the CD19^+^CD5^+^ cells within the lymphocytes.

## Supplementary Materials

The following are available online at https://www.mdpi.com/2077-0383/9/7/2312/s1: Figure S1: Spearman’s rank correlation analysis of the clinical parameters and the frequencies of the CD38-, CCR1-, and CCR2-expressing peripheral blood (PB) CD19^+^CD5^+^ lymphocytes in the newly diagnosed CLL patients.
