# Supplementary material for: Chemokine Receptors CCR1 and CCR2 on Peripheral Blood Mononuclear Cells of Newly Diagnosed Patients with the CD38-Positive Chronic Lymphocytic Leukemia
Source: J Clin Med. 2020 Jul 21;9(7):2312. doi: 10.3390/jcm9072312 (PMC7408836; doi:10.3390/jcm9072312)
Supplement: Supplementary file 1 [file jcm-09-02312-s001.pdf]

Supplementary Materials: Chemokine Receptors CCR1 and CCR2 on Peripheral Blood Mononuclear Cells of Newly Diagnosed Patients with the CD38-Positive Chronic Lymphocytic Leukemia

Irina Kholodnyuk, Alla Rivkina, Laura Hippe, Simons Svirskis, Svetlana Kozireva, Ildze Ventina, Irina Spaka, Marina Soloveichika, Jelena Pavlova, Modra Murovska and Sandra Lejniece

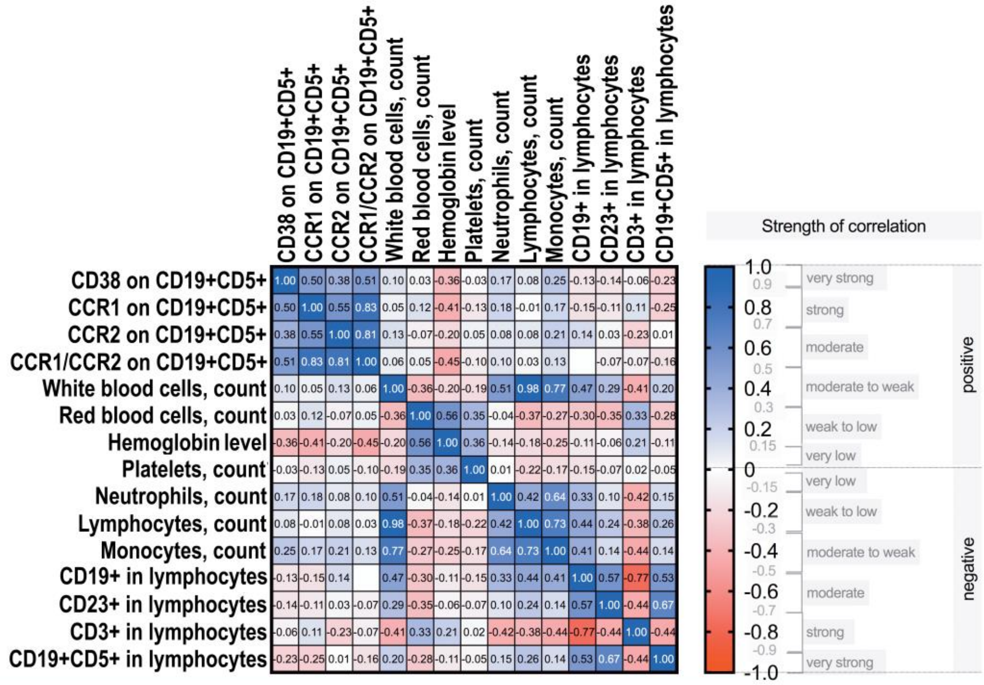

P-values	CD38 on CD19+CD5+	CCR1 on CD19+CD5+	CCR2 on CD19+CD5+	CCR1/2 on CD19+CD5+	White blood cells	Red blood cells	Hemoglobin level	Platelets, count	neu (neutrophils)	lym (lymphocytes)	mon (monocytes)	CD19+ in lymph.	CD23+ in lymph.	CD3+ in lymph.	CD19+CD5+ in lymph.
CD38 on CD19+CD5+		<0.0001	0.002	<0.0001	0.5	0.8	0.008	0.8	0.2	0.6	**0.08**	0.3	0.3	0.6	**0.07**
CCR1 on CD19+CD5+	<0.0001		<0.0001	<0.0001	0.7	0.4	0.002	0.4	0.2	0.9	0.2	0.2	0.4	0.4	**0.054**
CCR2 on CD19+CD5+	0.002	<0.0001		<0.0001	0.3	0.6	0.2	0.7	0.6	0.6	0.1	0.3	0.8	**0.07**	1.0
CCR1/2 on CD19+CD5+	<0.0001	<0.0001	<0.0001		0.7	0.7	0.001	0.5	0.5	0.9	0.4	1.0	0.6	0.6	0.2
White blood cells	0.5	0.7	0.3	0.7		0.008	0.2	0.2	<0.0001	<0.0001	<0.0001	0.0003	0.04	0.002	0.1
Red blood cells	0.8	0.4	0.6	0.7	0.008		<0.0001	0.01	0.8	0.01	**0.054**	0.03	0.01	0.02	0.04
Hemoglobin level	0.008	0.002	0.2	0.001	0.2	<0.0001		0.01	0.3	0.2	**0.08**	0.4	0.7	0.1	0.4
Platelets, count	0.8	0.4	0.7	0.5	0.2	0.01	0.01		1.0	0.1	0.2	0.3	0.6	0.9	0.7
neu (neutrophils)	0.2	0.2	0.6	0.5	0.0001	0.8	0.3	1.0		0.002	<0.0001	0.02	0.5	0.002	0.3
lym (lymphocytes)	0.6	0.9	0.6	0.8	<0.0001	0.01	0.2	0.1	0.002		<0.0001	0.001	**0.09**	0.006	**0.06**
mon (monocytes)	**0.08**	0.2	0.1	0.4	<0.0001	**0.054**	**0.08**	0.2	<0.0001	<0.0001		0.003	0.4	0.001	0.3
CD19+ in lymph.	0.3	0.2	0.3	1.0	<0.0001	0.03	0.4	0.3	0.02	0.001	0.003		<0.0001	<0.0001	<0.0001
CD23+ in lymph.	0.3	0.4	0.8	0.6	0.04	0.01	0.7	0.6	0.5	**0.09**	0.4	<0.0001		0.001	<0.0001
CD3+ in lymph.	0.6	0.4	**0.07**	0.6	0.002	0.02	0.1	0.9	0.002	0.006	0.001	<0.0001	0.001		0.0005
CD19+CD5+ in lymph.	**0.07**	**0.054**	1.0	0.2	0.1	0.04	0.4	0.7	0.3	**0.06**	0.3	<0.0001	<0.0001	0.0005	

Figure S1. Spearman's rank correlation analysis of the clinical parameters and the frequencies of the CD38-, CCR1-, and CCR2-expressing peripheral blood (PB) $CD19^{+}CD5^{+}$ lymphocytes in the newly diagnosed CLL patients. The Spearman's rank correlation analysis matrix is shown. CD38 on $CD19^{+}CD5^{+}$, the frequency of the CD38-expressing cells within the PB $CD19^{+}CD5^{+}$ lymphocytes; CCR1 on $CD19^{+}CD5^{+}$ and CCR2 on $CD19^{+}CD5^{+}$, the frequencies of the corresponding receptor-expressing cells within the PB $CD19^{+}CD5^{+}$ lymphocytes; CCR1/CCR2 on $CD19^{+}CD5^{+}$, the frequency of the CCR1- or CCR2-expressing cells within the PB $CD19^{+}CD5^{+}$ lymphocytes. The clinical parameters shown in Table 1 were analyzed. In the table, the corresponding *p*-values are presented. Note the *p*-values that are not exceeding the significance cut-off value of 0.05.
